# Prediction of well-being and insight into work-life integration among physicians using machine learning approach

**DOI:** 10.1371/journal.pone.0254795

**Published:** 2021-07-15

**Authors:** Masahiro Nishi, Michiyo Yamano, Satoaki Matoba

**Affiliations:** 1 Department of Cardiovascular Medicine, Graduate School of Medical Science, Kyoto Prefectural University of Medicine, Kyoto, Japan; 2 Cardiovascular Branch, National Heart, Lung and Blood Institute, National Institutes of Health, Bethesda, Maryland, United States of America; University of Toronto, CANADA

## Abstract

There has been increasing interest in examining physician well-being and its predictive factors. However, few studies have revealed the characteristics associated with physician well-being and work-life integration using a machine learning approach. To investigate predictive factors of well-being and obtain insights into work-life integration, the survey was conducted by letter mail in a sample of Japanese physicians. A total of 422 responses were collected from 846 physicians. The mean age was 47.9 years, males constituted 83.3% of the physicians, and 88.6% were considered to be well. The most accurate machine learning model showed a mean area under the curve of 0.72. The mean permutation importance of career satisfaction, work hours per week, existence of family support, gender, and existence of power harassment were 0.057, 0.022, 0.009, 0.01, and 0.006, respectively. Using a machine learning model, physician well-being could be predicted. It seems to be influenced by multiple factors, such as career satisfaction, work hours per week, family support, gender, and power harassment. Career satisfaction has the highest impact, while long work hours have a negative effect on well-being. These findings support the need for organizational interventions to promote physician well-being and improve the quality of medical care.

## Introduction

Physician well-being extends beyond individual issues and has a significant impact on healthcare systems and patient care. Unwell physicians could contribute to low quality of care, lapses in professionalism, and jeopardizing patient safety [1–6]. Demographic characteristics such as gender, age, and relationship status are reported to be associated with physician well-being [7]. Physician well-being is not merely the absence of distress, but also comprises being challenged, thriving, and achieving success in various kinds of personal and social aspects [8]. It is a complex and multifactorial issue.

Problems with work-life integration and burnout are common among physicians [9–11] and physician assistants [12]. As current medical practice demands more time, physicians are more likely to be fatigued, stressed, or burned out [13–15]. Physicians specializing in emergency medicine and primary care have a higher risk of burnout [16–20]. Among Japanese physicians working in stroke care units, heavy workload, short sleep duration, little experience, and low mental quality of life were determined to be the risk factors for burnout [21]. A systematic review shows that burnout or stress in physicians is predominantly associated with workplace-related factors, such as work demands and poor work environment, rather than non-modifiable and non-workplace-related factors [22].

Limited research has been conducted to identify the factors determining well-being and work-life integration among physicians, despite the abundant reports about physician burnout. To our knowledge, there are no reports using a machine learning approach to study physician well-being. To bridge this gap in the literature, a survey was conducted among 846 physicians who belonged to a union of the Department of Second Internal Medicine at Kyoto Prefectural University of Medicine in Japan. In this study, we reveal that the machine learning approach is feasible for predicting physician well-being, and variables such as career satisfaction and work hours per week are important predictive factors for physician well-being.

## Methods

A letter mail survey was conducted among 846 physicians, including cardiologists, nephrologists, pulmonologists, hematologists, gastroenterologists, and family doctors (S1 Fig) in a union of the Department of Second Internal Medicine at Kyoto Prefectural University of Medicine in Japan, which was established in 1921. The participants responded to the survey by mail. They volunteered to participate, informed consent was obtained by written form before filling out the survey, and all responses remained anonymous. The survey was conducted between March 1 and 31, 2019. The data analysis took place from November 1, 2019, to March 30, 2020. A total of 422 responses were collected, yielding a participation rate of 49.9%. The characteristics of total survey subjects and survey respondents were shown in S2 Fig. Of the respondents, 63 with no work hours were excluded because they were considered to have retired. The protocol for the present study was approved by the committee of the graduates’ association of Kyoto Prefectural University of Medicine. The entire protocol of the present study was designed in accordance with the Declaration of Helsinki.

### Study measures

The primary independent variable in the present study was well-being, which was assessed on a scale of 1 to 10. Physicians were considered to be well when they reported a high score of 6 to 10 and unwell when they reported a low score of 1 to 5. Age, gender, relationship status, work, work style, work hours per week, career satisfaction, family support, sexual harassment, power harassment, equality at home, and equality at the workplace were the 12 variables considered (S1 Appendix). The variables were used as categorical values (well-being, gender, relationship status, work, work style, career satisfaction, family support, sexual harassment, power harassment, equality at home, and equality at the workplace) or numeric values (age and work hours per week).

### Primary analysis

An unwell state of well-being was set as the target of prediction modeling. An ensemble model of machine learning, comprising elastic net (ENET), average (AVG), median (MED), and generalized linear model (GLM) blender, was used for the dual classification of well-being (S2 Appendix). To avoid overfitting, 10-fold cross validation and 10 seeds randomization were performed. The performance of each model was evaluated and compared using the validation data set (10% of sample). AUC was used as an indicator of the model accuracy. Permutation importance was calculated for variable impact on target, according to previous studies [23,24]. Mutual information was calculated for each variable. Low-impact variables were excluded at each round when the minimum permutation importance was lower than 0; consequently, a total of 5 rounds were conducted. Partial dependence was calculated to evaluate the effect of each variable on the target. Analysis and modeling were performed using Python 3.7.4, with DataRobot 2.21.3 being deployed.

## Results

### Sample characteristics

Data on participant characteristics associated with well-being and work-life integrity were available for 359 physicians (S3 Appendix), of whom 88.6% [318 of 359] were found to be well; their mean [SD] age was 47.9 [14.8] years; 83.3% [299 of 359] of them were male; 64.9% [233 of 359] were hospital workers and 91.9% [330 of 359] were full time workers. Their mean [SD] work hours per week were 45.5 [17.6]. A majority of them, 90.8% [326 of 359], were married; 37.6% [135 of 359] had family support; 48.7% [175 of 359] enjoyed equality at work; and 41.8% [150 of 359] enjoyed equality at home. However, 74.4% [267 of 359] of the physicians had suffered power harassment and 52.9% [190 of 359] had experienced sexual harassment. Despite this, the majority, 72.1% [259 of 359], enjoyed career satisfaction (Table 1). Work hours per week and career satisfaction were significantly different between unwell and well state (S4 Appendix).

**Table 1:** Participant characteristics associated with well-being and work–life integration.

Variable		Data type	No. (%)
Well-being		Categorical	
	Well		318 (88.6)
	Unwell		41 (11.4)
Age		Numeric	
	Mean (SD)		47.9 (14.8)
Gender		Categorical	
	Male		299 (83.3)
	Female		60 (16.7)
Work		Categorical	
	Hospital worker		233 (64.9)
	Practitioner		126 (35.1)
Work style		Categorical	
	Full-time		330 (91.9)
	Part-time		29 (8.1)
Work hours per week		Numeric	
	Mean (SD)		45.5 (17.6)
Relationship status		Categorical	
	Married		326 (90.8)
	Single		33 (9.2)
Family support		Categorical	
	Yes		135 (37.6)
	No		224 (62.4)
Equality at work		Categorical	
	Yes		175 (48.7)
	No		184 (51.3)
Equality at home		Categorical	
	Yes		150 (41.8)
	No		209 (58.2)
Power harassment		Categorical	
	Yes		267 (74.4)
	No		92 (25.6)
Sexual harassment		Categorical	
	Yes		190 (52.9)
	No		169 (47.1)
Career satisfaction		Categorical	
	Yes		259 (72.1)
	No		100 (27.9)

### Machine-learning models and variable importance

**Fig 1:**
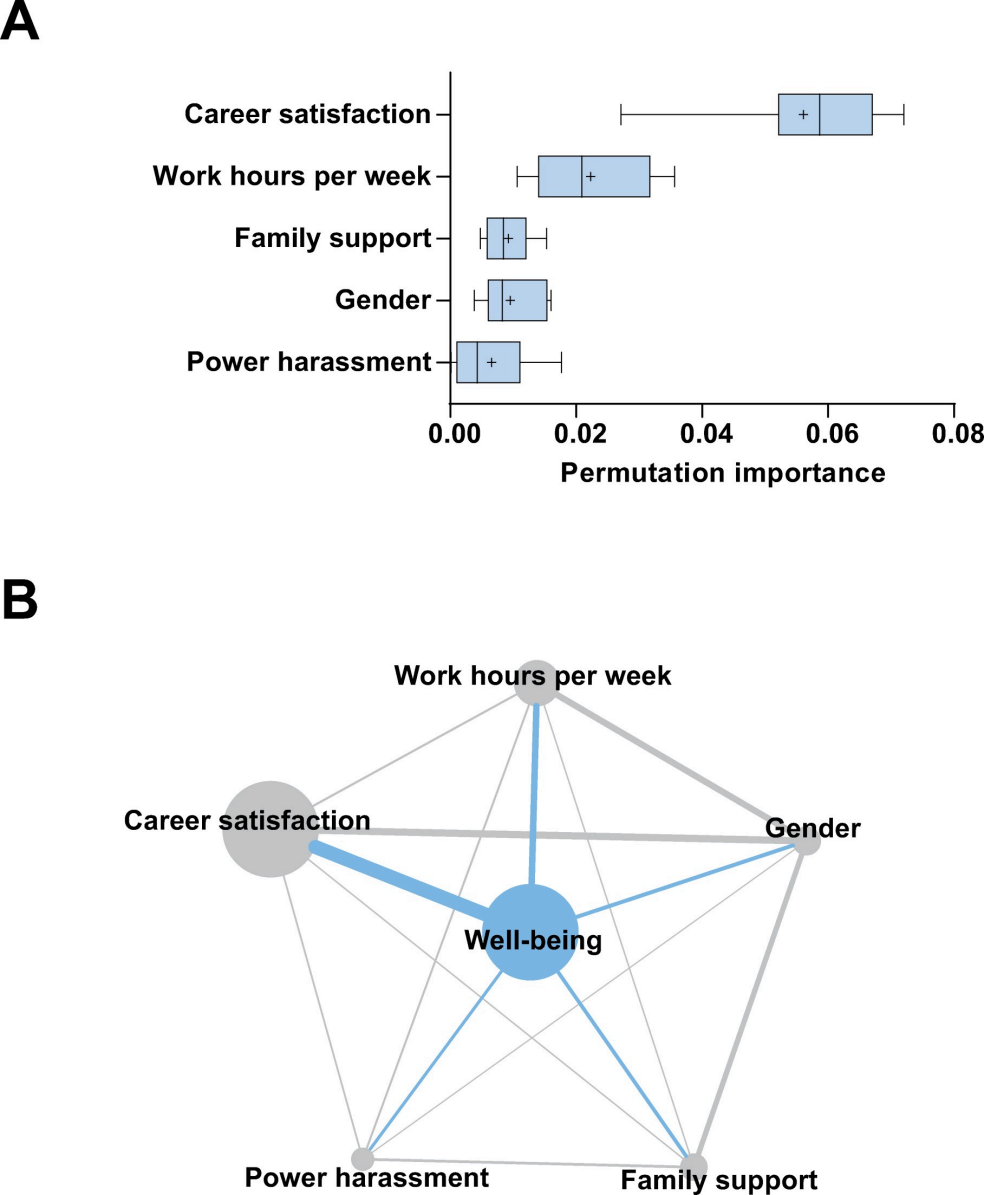
Variable impact on physician well-being. (**A**) Permutation importance on physician well-being was calculated for selected covariates: career satisfaction, work hours per week, family support, gender, and power harassment. Boxplots represents median, interquartile range, and extreme values. + indicates mean value. (**B**) Network between factors associated with well-being. The network between well-being and each variable is depicted in blue; the edge width and node size is determined according to the permutation importance of each variable. The network between variables is depicted in gray; the edge width is determined based on the mutual information between the two variables.

**Fig 2:**
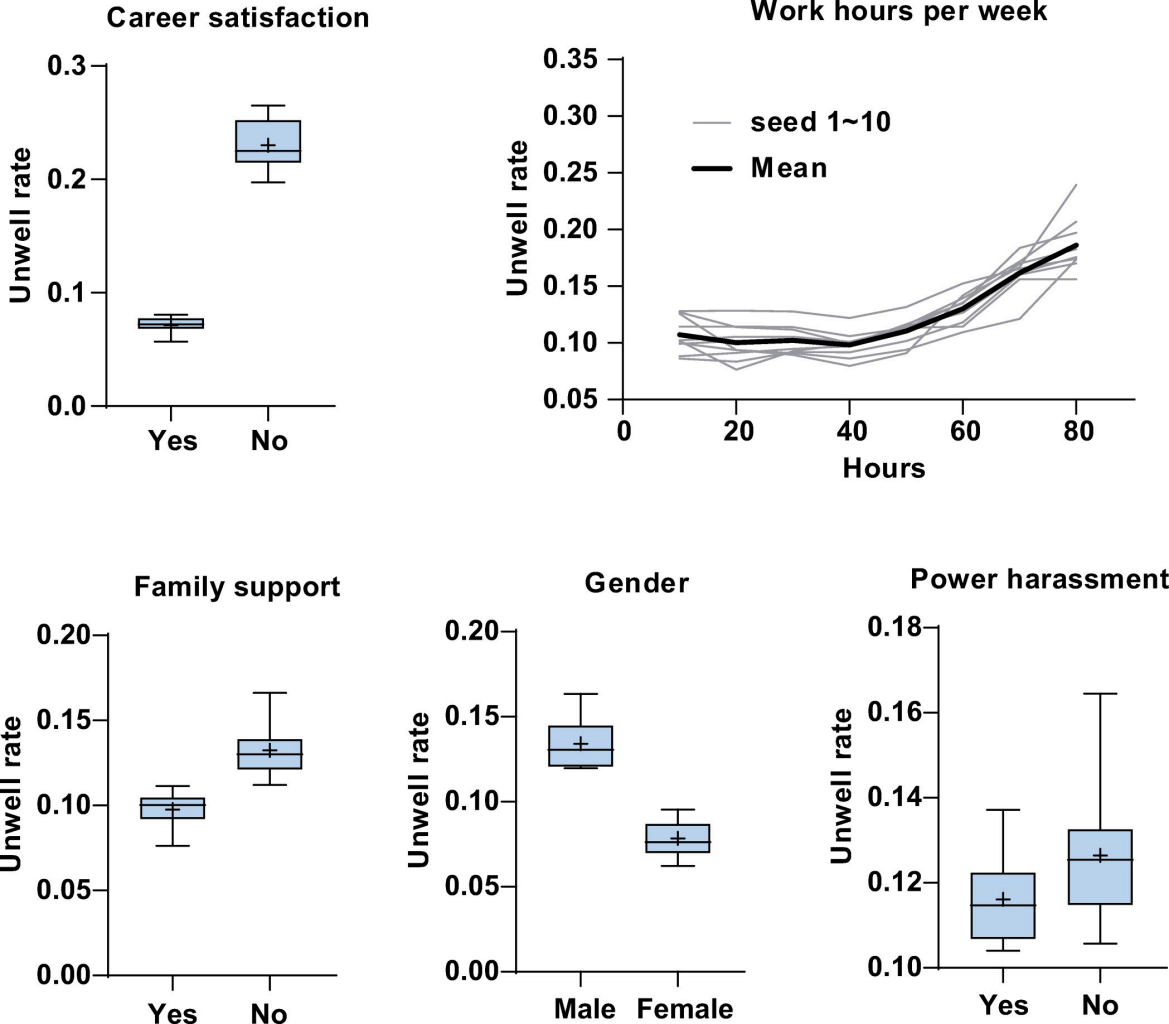
Effect of variable on physician well-being. Partial dependence on physician unwell state was shown for selected covariates: career satisfaction, work hours per week, family support, gender, and power harassment. Boxplots represents median, interquartile range, and extreme values. + indicates mean value. Unwell rate indicates predicted probability of unwell state.

The mean [SD] area under the curve (AUC) was 0.70 [0.02] in round 1, where all 12 variables were used for prediction modeling. In round 2, excluding work and work style, the mean [SD] AUC was 0.70 [0.02]. In round 3, excluding equality at work and relationship status, the mean [SD] AUC was 0.70 [0.02]. In round 4, excluding the existence of sexual harassment and equality at home, the mean [SD] AUC was 0.69 [0.02]. Excluding age, the model in round 5 with the remaining five covariates showed the highest accuracy: mean [SD] AUC 0.72 [0.02] (Table 2). Therefore, the remaining five variables were further examined. Several regression models were also evaluated to predict well-being index (1–10); however, those accuracy were not sufficient compared to binary classification models (S5 Appendix). The mean [SD] permutation importance of career satisfaction, work hours per week, family support, gender, and power harassment were 0.057 [0.012], 0.022 [0.009], 0.009 [0.003], 0.01 [0.005], and 0.006 [0.006], respectively. Among them, career satisfaction had the highest impact, with work hours per week also showing a high impact on well-being (Fig 1A). The network between factors associated with well-being was depicted according to the mutual information and the permutation importance of each variable using Cytoscape 3.6.1 (Fig 1B) [25]. The partial dependence of each variable is shown in Fig 2. The difference in the mean unwell rate was 16% between participants with or without career satisfaction. Long work hours per week were correlated with the unwell rate. The difference in the mean unwell rate was 3.5% between participants with or without family support. The difference in the mean unwell rate was 5.6% between males and females. Consistent with low permutation importance, the partial dependence of power harassment seemed indistinct due to its large error. To exclude the influence of work style as a possible cofounder, the data excluding part-time worker was also analyzed, then permutation importance for each variable was calculated (S3 Fig). The work hours per week showed similar importance on well-being to all data analysis.

**Table 2:** Model accuracy of each round and random seed.

	Round 1		Round 2		Round 3		Round 4		Round 5	
Seed No.	Model	AUC	Model	AUC	Model	AUC	Model	AUC	Model	AUC
Seed 1	MED blender	0.69	MED blender	0.70	ENET blender	0.70	MED blender	0.692	MED blender	0.70
Seed 2	AVG blender	0.69	AVG blender	0.69	AVG blender	0.69	MED blender	0.695	MED blender	0.71
Seed 3	MED blender	0.70	MED blender	0.71	AVG blender	0.70	ENET blender	0.699	ENET blender	0.69
Seed 4	ENET blender	0.75	ENET blender	0.72	MED blender	0.72	ENET blender	0.684	MED blender	0.74
Seed 5	ENET blender	0.69	GLM blender	0.71	GLM blender	0.76	MED blender	0.703	MED blender	0.72
Seed 6	AVG blender	0.70	AVG blender	0.72	AVG blender	0.71	AVG blender	0.702	AVG blender	0.71
Seed 7	GLM blender	0.70	AVG blender	0.70	MED blender	0.68	ENET blender	0.712	MED blender	0.75
Seed 8	AVG blender	0.68	AVG blender	0.68	MED blender	0.69	AVG blender	0.658	AVG blender	0.71
Seed 9	MED blender	0.70	MED blender	0.69	AVG blender	0.70	AVG blender	0.666	AVG blender	0.71
Seed 10	MED blender	0.69	GLM blender	0.73	ENET blender	0.69	ENET blender	0.686	GLM blender	0.75
Mean (SD)		0.70 (0.02)		0.70 (0.02)		0.70 (0.02)		0.69 (0.02)		0.72 (0.02)

AUC indicates area under the curve; ENET, elastic net; AVG, average; MED, median; GLM, generalized linear model.

## Discussion

Among Japanese physicians in this study, well-being was predicted by ensemble models of machine learning, and the determinants of work-life integration were evaluated. Model accuracy using AUC was sufficient to predict well-being by selecting five important covariates: career satisfaction, work hours per week, family support, gender, and power harassment. In particular, career satisfaction and work hours per week were high-impact predictive factors for well-being.

This study provided insights into the association between physician well-being and work-life integration. Career satisfaction was the highest-impact predictive factor for well-being. Physicians with burnout are more likely to experience not only career dissatisfaction [7,14,26] but also career choice regret among resident physicians [27]. Long work hours per week had a significant impact on well-being. Those who frequently work long shifts suffer negative consequences in terms of healthcare performance [28,29]. In addition, they have an increased risk of percutaneous injuries and motor vehicle accidents [30,31]. Moreover, physicians without family support had a higher partial dependence on the unwell rate despite its low permutation importance. Various kinds of harassment prevail in medical training [32]. Sexual harassment was excluded as a covariate because the permutation impact was low. Power harassment is a form of workplace harassment in which the someone in a greater position uses the power to harass or bully a victim who is lower on the office hierarchy. Power harassment was a low-impact predictive factor for well-being, with unexpected results of partial dependence. Although this survey included an array of doctors with varying specializations, specialty was not evaluated. There are also different types of hospital workers from the business management to work shift staff. This study showed no impact of such work differences between health practitioners and hospital workers in terms of well-being.

Demographic characteristics such as age, gender, and relationship status were evaluated in association with physician well-being. This survey was conducted only among Japanese physicians, although race/ethnicity is an independent predictive factor of physician burnout [33–35]. Age and relationship status were excluded as covariates because the permutation importance was low. Young trainee physicians are at high-risk for burnout and stress compared to older physicians [36–38]. Gender was a predictive factor, but it had a relatively low impact on well-being. Male physicians showed a higher partial dependence on the unwell rate than did female physicians. Another report revealed that female residents have a higher risk of burnout than do male residents [27]. In addition, female physicians are more likely to experience work–home conflicts than their male counterparts [9]. Moreover, many female physicians are subject to various hurdles such as male-dominant structures of medical society, unconscious discrimination, or unfair evaluation by medical directors [39,40]. Thus, consistent with a previous report [22], demographic and non-work place factors such as gender, age, and relationship status had a relatively lower impact on physician well-being in this study.

This study has several limitations. First, this survey was conducted in a single center for Japanese physicians. Second, the limited number of participants might have caused the study to be underpowered. The observational findings will need to be evaluated in nationwide survey. Third, some important factors associated with well-being, such as specialty, race, and income of physicians, were not assessed in this survey. Fourth, the evaluation measurement for subjective indicators might be difficult to reproduce in future studies. Fifth, the participation rate of 49.9% was not high. Sixth, the previous emotional state or medical history of participants, such as depression or posttraumatic stress disorder, was not investigated. A comparative study before and after COVID19 pandemic will be interesting.

In conclusion, physician well-being was predicted using a machine learning model. Career satisfaction, work hours per week, family support, gender, and power harassment were predictive factors for well-being. Career satisfaction had a significant impact, and long work hours per week had a negative effect on well-being. Further studies are needed to better understand these results and ensure improvements in physician well-being, work–life integration, and medical care quality.

## Supporting information

S1 Fig Specialty of total survey subjects. Distribution of each speciality is indicated by n (%).
(PPTX)

S2 Fig Characteristics of total survey subjects and survey respondents. **A**, Gender distribution. Distribution for each gender is indicated by n (%). **B**, Age distribution. Age for each gender is indicated by mean [SD].
(PPTX)

S3 Fig Permutation importance of each variable for the data excluding part-time worker. Ensemble models were used for the dual classification of well-being. Mean area under the curve (AUC) was used as an indicator of the model accuracy. Boxplots represent median, min,and max value.
(PPTX)

S1 Appendix Survey for physician well-being. (DOCX)

S2 Appendix Algorithm of machine learning model. (DOCX)

S3 Appendix All data set of the survey. (XLSX)

S4 Appendix Participant characteristics for unwell and well state. (DOCX)

S5 Appendix Accuracy of regression models for physician well-being. (DOCX)
