# Supplementary material for: Prediction of well-being and insight into work-life integration among physicians using machine learning approach
Source: PLoS One. 2021 Jul 15;16(7):e0254795. doi: 10.1371/journal.pone.0254795 (PMC8282024; doi:10.1371/journal.pone.0254795)
Supplement: S1 Fig — Distribution of each speciality is indicated by n (%). (PPTX) [file pone.0254795.s001.pptx]

## Slide 1
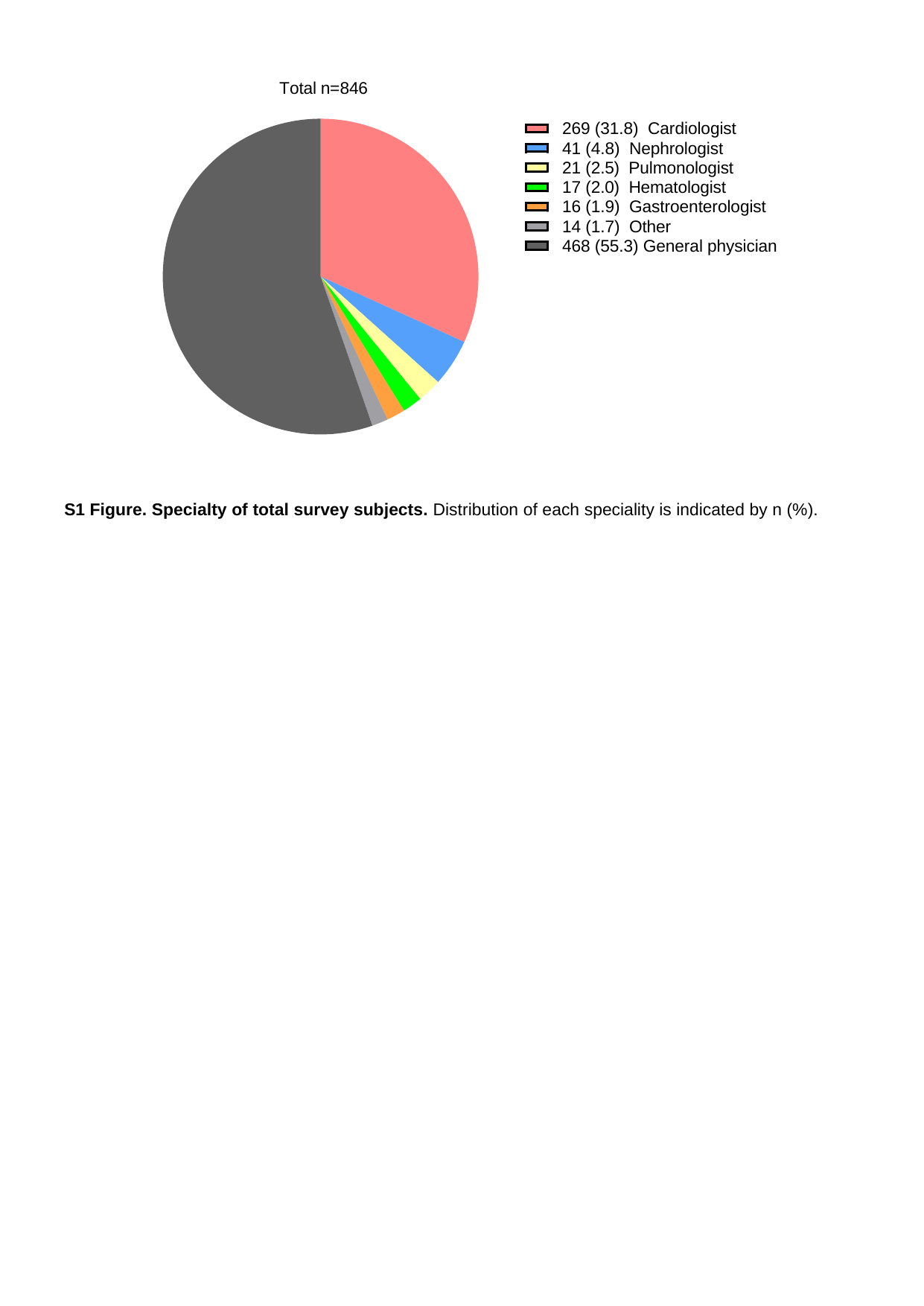

S1 Figure. Specialty of total survey subjects. Distribution of each speciality is indicated by n (%).
