# Supplementary material for: Prediction of well-being and insight into work-life integration among physicians using machine learning approach
Source: PLoS One. 2021 Jul 15;16(7):e0254795. doi: 10.1371/journal.pone.0254795 (PMC8282024; doi:10.1371/journal.pone.0254795)
Supplement: S2 Fig — A, Gender distribution. Distribution for each gender is indicated by n (%). B, Age distribution. Age for each gender is indicated by mean [SD]. (PPTX) [file pone.0254795.s002.pptx]

## Slide 1
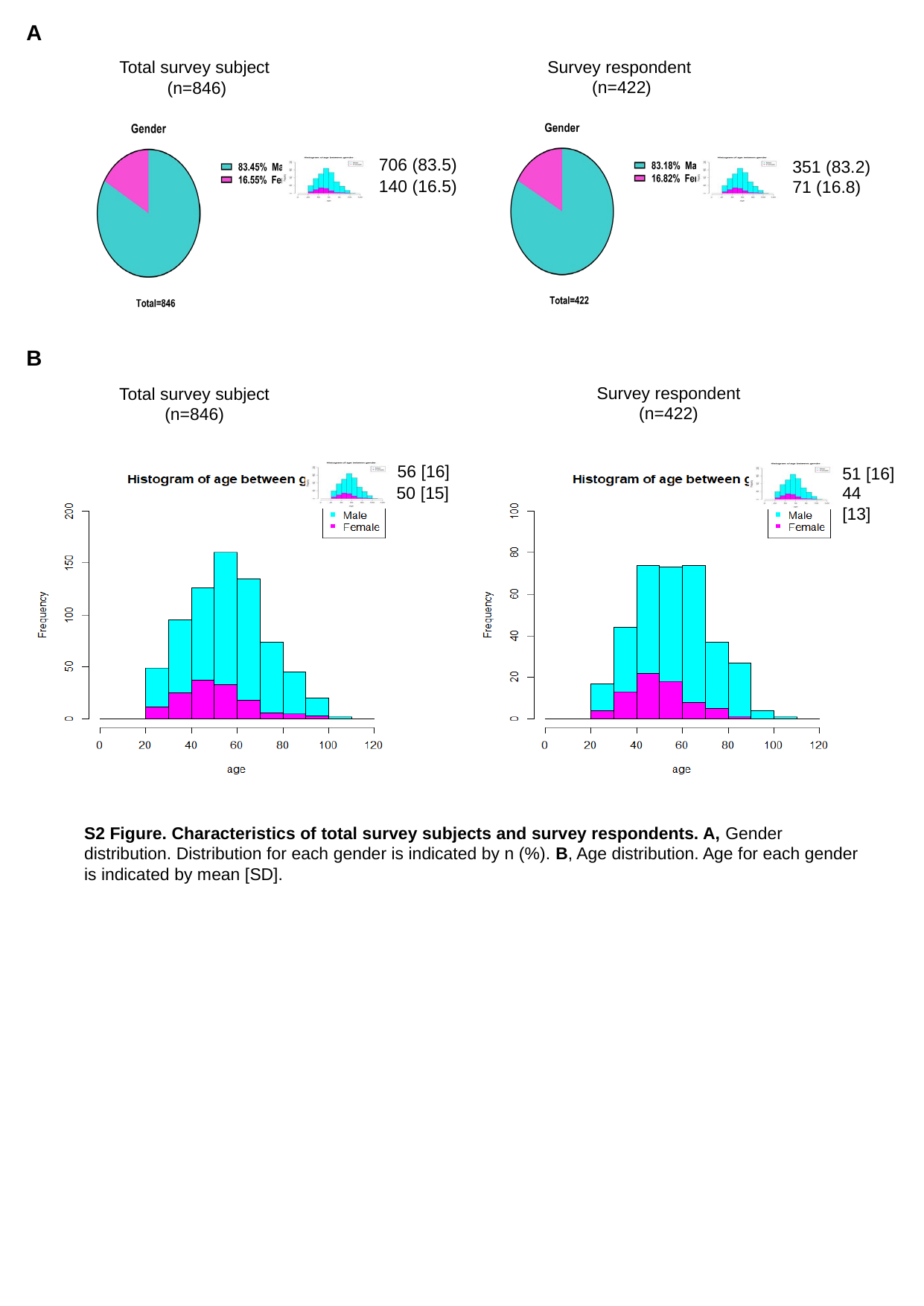

A
Survey respondent
(n=422)
Total survey subject
(n=846)
706 (83.5)
351 (83.2)
140 (16.5)
71 (16.8)
B
Survey respondent
(n=422)
Total survey subject
(n=846)
56 [16]
51 [16]
50 [15]
44 [13]
S2 Figure. Characteristics of total survey subjects and survey respondents. A, Gender distribution. Distribution for each gender is indicated by n (%). B, Age distribution. Age for each gender is indicated by mean [SD].
