# Supplementary material for: Prediction of well-being and insight into work-life integration among physicians using machine learning approach
Source: PLoS One. 2021 Jul 15;16(7):e0254795. doi: 10.1371/journal.pone.0254795 (PMC8282024; doi:10.1371/journal.pone.0254795)
Supplement: S3 Fig — Ensemble models were used for the dual classification of well-being. Mean area under the curve (AUC) was used as an indicator of the model accuracy. Boxplots represent median, min,and max value. (PPTX) [file pone.0254795.s003.pptx]

## Slide 1
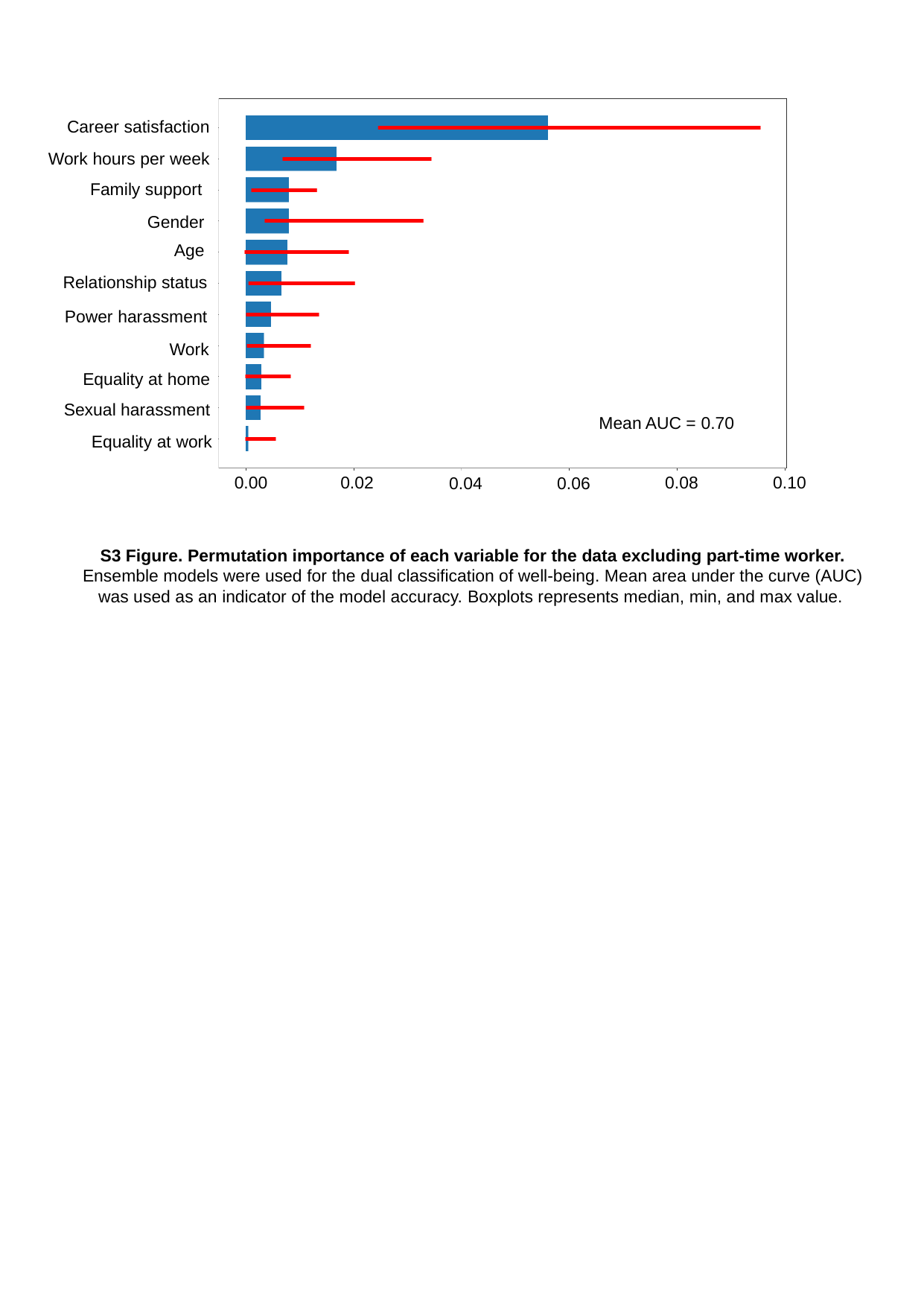

Career satisfaction
Work hours per week
Family support
Gender
Age
Relationship status
Power harassment
Work
Equality at home
Sexual harassment
Mean AUC = 0.70
Equality at work
0.10
0.08
0.00
0.02
0.06
0.04
S3 Figure. Permutation importance of each variable for the data excluding part-time worker. Ensemble models were used for the dual classification of well-being. Mean area under the curve (AUC) was used as an indicator of the model accuracy. Boxplots represents median, min, and max value.
