# Supplementary material for: Prediction of well-being and insight into work-life integration among physicians using machine learning approach
Source: PLoS One. 2021 Jul 15;16(7):e0254795. doi: 10.1371/journal.pone.0254795 (PMC8282024; doi:10.1371/journal.pone.0254795)
Supplement: S1 Appendix — (DOCX) [file pone.0254795.s004.docx]

**S1 Appendix. Survey for physician well-being**

**Survey for physician well-being**

**1. Age**

1. 20s 2. 30s 3. 40s 4. 50s 5. 60s

6. 70s 7. 80s 8. 90s 9. 100s -

**2. Gender**

1. Male 2. Female

**3. Relationship status**

1. Married 2. Single

**4. Work**

1. Hospital worker 2. Practitioner

**5. Work style**

1. Full-time 2. Part-time

**6. Work hours per week**

1. 0 h 2. < 10 h

3. < 20 h 4. < 30 h

5. < 40 h 6. < 50 h

7. < 60 h 8. < 70 h

9. < 80 h 10. 80 h ≦

**7. Are you satisfied with your career?**

1. Yes 2. No

**8. Do you have family support?**

1. Yes 2. No

**9. Have you experienced sexual harassment in work place?**

1. Yes 2. No

**10. Have you experienced power harassment in work place?**

1. Yes 2. No

**11. Do you feel equality at home?**

1. Yes 2. No

**12. Do you feel equality at work?**

1. Yes 2. No

**13. Well-being**

Unwell (1 2 3 4 5 6 7 8 9 10) Very well

Unwell: 1-5, Well: 6-10

**Japanese version**

**医師のwell-beingに関する調査**

**1. 年齢をお答えください**

1．20歳代 2．30歳代　　3．40歳代　　4．50歳代　　 5．60歳代

6．70歳代 　7．80歳代　 8．90歳代　　9．100歳代以上

**2. 性別をお答えください**

1．男性　　　　2．女性

**3. 結婚はされていますか**

1．未婚　　　 2．既婚

**4. 勤務先をお答えください**

1．開業　　　　2．病院勤務

**5. 勤務形態をお答えください**

1．常勤　　　　2．非常勤

**6. 一週間当たりの勤務時間をお答えください**

1．0時間　　　　　　　　　　 2．10時間未満

3．20時間未満　　　　 　　　 4．30時間未満

5．40時間未満　　　　　　　 6．50時間未満

7．60時間未満　　 　 8．70時間未満

9．80時間未満　　　　 10．80時間以上

**7. キャリアに満足していますか**

1．はい　　　　　2．いいえ

**8. 家族の協力がありますか**

　 1．はい　　 2．いいえ

**9. 職場でセクシャルハラスメントを受けたことがありますか**

1．はい　　　　　2．いいえ

**10. 職場でパワーハラスメントメントを受けたことがありますか**

1．はい　　　　　2．いいえ

**11. 家庭において平等感を感じますか**

1．はい　　　　　2．いいえ

**12. 職場において平等感を感じますか**

1．はい　　　　　2．いいえ

**13. 現在のご自身のwell-beingはどの段階に相当しますか**

幸福ではない (1 2 3 4 5 6 7 8 9 10) 非常に幸福

幸福ではない: 1-5, 幸福: 6-10
