# Supplementary material for: Prediction of well-being and insight into work-life integration among physicians using machine learning approach
Source: PLoS One. 2021 Jul 15;16(7):e0254795. doi: 10.1371/journal.pone.0254795 (PMC8282024; doi:10.1371/journal.pone.0254795)
Supplement: S2 Appendix — (DOCX) [file pone.0254795.s005.docx]

**S2 Appendix. Algorithm of machine learning model**

We created robust and accurate ensemble model by combining independent models created from multiple independent algorithms, using Python 3.7.4, with DataRobot 2.21.3 being deployed. The following steps were performed:

**Random seeding**

**&**

**CV partitioning**

**Machine learning modelling**

AVG blender

MED blender

ENET blender

GLM blender

**Model performance evaluation**

**Variable selection**

**1.** The random seed controlling the random sampling condition in cross-validation (CV) partitioning was changed 10 times.

**2.** 10-fold CV was conducted with 0% holdout. (Partitioning employs stratified extraction so that the ratio of true/false is the same for all folds.)

**3.** Multiple ensemble models were generated; single machine learning models with different algorithmic predispositions (e.g., eXtreme gradient-boosted trees, random forest, regularized regression such as Elastic Net, and Neural Networks) were combined. The ensembles also applied various methods such as average (AVG), median (MED), elastic net (ENET), and generalized linear model (GLM) blender.

**4.** Model performance was evaluated by area under the curve of the receiver operating characteristic curve.

**5.** Permutation importance was calculated for the most accurate ensemble. Since going through these steps finally yielded 10 permutation importance values for each explanatory variable, its mean, median, interquartile range, and extreme values were calculated. Then variable selection was performed to ensure that no explanatory variables with relatively small values were included in the model. We repeated the above steps to narrow down the results to only the important variables.

**6.** To understand the independent impact of individual variables on target, we constructed a partial dependence plot. The partial dependence plot can be interpreted as showing the effect of changing a variable in isolation; it demonstrates the relationship between the value of that variable and the probability value of the target.
