# Supplementary material for: Prediction of well-being and insight into work-life integration among physicians using machine learning approach
Source: PLoS One. 2021 Jul 15;16(7):e0254795. doi: 10.1371/journal.pone.0254795 (PMC8282024; doi:10.1371/journal.pone.0254795)
Supplement: S4 Appendix — (DOCX) [file pone.0254795.s007.docx]

**S4 Appendix. Participant characteristics for unwell and well state.**

| **Variable** | **Unwell (1-5)**  **(n=41)** | **Well (6-10)**  **(n=318)** | **P-value** |
| --- | --- | --- | --- |
| Age | 45.9±14.1 | 48.2±14.9 | 0.21 |
| Gender (Female) | 4 (9.8%) | 56 (17.6%) | 0.2 |
| Work (Hospital worker) | 29 (70.7%) | 204 (64.2%) | 0.4 |
| Work style (Full-time) | 38 (92.7%) | 292 (91.8%) | 0.85 |
| Work hours per week | 50.0±20.7 | 44.9±17.1 | 0.04 |
| Relationship status (Married) | 34 (82.9%) | 292 (91.8%) | 0.06 |
| Family support (Yes) | 12 (29.3%) | 123 (38.7%) | 0.24 |
| Equality at work (Yes) | 20 (48.8%) | 155 (48.7%) | 0.99 |
| Equality at home (Yes) | 17 (41.5%) | 133 (41.8%) | 0.96 |
| Power harassment (Yes) | 31 (75.6%) | 236 (74.2%) | 0.84 |
| Sexual harassment (Yes) | 22 (53.7%) | 168 (52.8%) | 0.92 |
| Career satisfaction (Yes) | 16 (39.0%) | 243 (76.4%) | <0.01 |

Chi-square test or student’s t-test was performed for the statistical analysis.
