# Supplementary material for: Prediction of well-being and insight into work-life integration among physicians using machine learning approach
Source: PLoS One. 2021 Jul 15;16(7):e0254795. doi: 10.1371/journal.pone.0254795 (PMC8282024; doi:10.1371/journal.pone.0254795)
Supplement: S5 Appendix — (DOCX) [file pone.0254795.s008.docx]

| **Metrix** | **Model** | | |
| --- | --- | --- | --- |
|  | **XGB** | **LGB** | **Ridge** |
| R^2^ | 0.038 | 0.025 | 0.022 |
| RMSE | 1.55 | 1.56 | 1.57 |
| MAE | 1.2 | 1.22 | 1.22 |

**S5 Appendix. Accuracy of regression models for physician well-being**

Machine learning modelling were performed with all 12 variables. Ridge, Ridge Regressor; XGB, eXtreme Gradient Boosted Trees Regressor; LGB, Light Gradient Boosted Trees Regressor; RMSE, Root-mean-square deviation; MAE, mean absolute error.
